# The Efficacy and Safety of Rituximab for Childhood Steroid-Dependent Nephrotic Syndrome: A Systematic Review and Meta-Analysis

**DOI:** 10.3389/fped.2021.728010

**Published:** 2021-08-20

**Authors:** Sidi Liu, Chuying Gui, Zhenzhen Lu, Huijie Li, Zhike Fu, Yueyi Deng

**Affiliations:** Department of Nephrology, Longhua Hospital, Shanghai University of Traditional Chinese Medicine, Shanghai, China

**Keywords:** rituximab, childhood steroid-dependent nephrotic syndrome, efficacy, safety, meta-analysis

## Abstract

**Objectives:** Rituximab (RTX), a possible alternative treatment option, is recognized as a new therapeutic hope for the treatment of steroid-dependent nephrotic syndrome (SDNS) in children. However, the efficacy and safety of RTX in the treatment of childhood SDNS are still controversial. The objective of this study was to evaluate the efficacy and safety of RTX treatment in children with SDNS.

**Study Design:** Six randomized controlled trials (RCTs) and one retrospective comparative control study data from studies, performed before January 2021 were collected, from PubMed, Cochrane Library, Embase, and Web of Science. The studies evaluating the efficacy and safety of RTX in childhood SDNS were included.

**Results:** Six RCTs and one retrospective comparative control study were included in our analysis. Compared with the control group, the RTX treatment group achieved a higher complete remission rate (OR = 5.21; 95% CI, 3.18–8.54; *p* < 0.00001), and we found significant differences between the two groups on serum albumin level (MD = 0.88; 95% CI, 0.43–1.33; *p* = 0.0001) and estimated glomerular filtration rate (MD = 6.43; 95% CI, 2.68–10.19; *p* = 0.0008). However, RTX treatment did not significantly lower serum creatinine levels nor did it significantly reduce the occurrence of proteinuria. In addition, we found no advantages with RTX on treatment safety.

**Conclusions:** RTX has shown satisfactory characteristics in terms of efficacy and may be a promising treatment method for SDNS in children. However, the long-term effects have not been fully evaluated and should be further studied through randomized clinical trials.

## Introduction

Nephrotic syndrome (NS) is a common and multiple glomerular disease in pediatrics. The main clinical features are massive proteinuria, hypoalbuminemia, hyperlipidemia, and edema (1). NS occurs in 16 out of every 100,000 children (2), and it brings greater financial and mental pressure to patients and their families. If it is not controlled in time, serious complications may occur. The development of end-stage renal disease seriously affects the quality of life of children (3). Among them, 75% of children with NS have minimal change nephropathy, which is sensitive to hormone therapy but easily leads to relapse and hormone dependence. Such children often need to extend the hormone medication time or add other immunosuppressants and cell-poisonous drugs (4). Commonly used immunosuppressants include cyclophosphamide, cyclosporine A, tacrolimus, and mycophenolate mofetil. Although the efficacy of these medications is acceptable, their associated adverse events and toxicities would limit their use in long-term maintenance therapy, such as growth and development restriction, weakened immune function, nephrotoxicity, and dyslipidemia (5). Therefore, new drugs are needed to solve these problems.

Rituximab (RTX) is a chimeric monoclonal antibody that targets the transmembrane protein CD20 on B lymphocytes. It was initially effective in the treatment of B-cell lymphoma, and then used to treat diseases like systemic lupus erythematosus, rheumatoid arthritis, and vasculitis (6). In recent years, scholars from many countries have used RTX as a treatment drug for children with NS and have achieved certain effects in treating childhood nephrotic syndrome (7–9). However, the efficacy and safety of RTX for this disease are still controversial, and the mechanism of action and safety in the disease are still unclear. It is still a matter of debate on the treatment of patients with childhood steroid-dependent nephrotic syndrome (SDNS). Thus, we conducted a meta-analysis of the efficacy and safety of RTX in the treatment of childhood SDNS.

## Methods

### Information Sources and Search Strategy

This meta-analysis was conducted in accordance with the Preferred Reporting Items for Systematic Reviews and Meta-Analyses statement (10). The search strategy was performed in the digital databases of PubMed, Cochrane Library, Embase, and Web of Science from their inception dates to January 2021. Two investigators independently performed a systematic search using the following search terms: “rituximab,” “CD20,” “children,” and “nephrotic syndrome,” at the same time, backtracking search for references of related literature.

### Study Selection and Data Collection Process

The initial assessment was based on screening the titles and abstracts; two independent reviewers excluded irrelevant documents based on the inclusion and exclusion criteria. Studies that were not excluded after the initial evaluation were screened in full text, and whether to be included in our analysis was determined according to the inclusion criteria. If there is a disagreement, it is up to the authors to reach a consensus and make the final decision. Case reports, review articles, meeting abstracts, comments, and studies containing mixed pediatric and adult populations without subgroup analysis were excluded.

We extracted patients' outcomes that comprised complete remissions, serum albumin, serum creatinine, proteinuria, eGFR, and related adverse events. Data extraction was done by two independent reviewers, including authors, publication year, country where the study was conducted, study design, sample size, age, sex, interventions, study outcomes, the follow-up, and adverse events, and outcomes with incomplete data were excluded from the analysis.

### Bias and Quality Assessments of the Included Studies

Each quality of the RCT study was assessed according to the “risk of bias” of the Cochrane Collaboration, which includes random sequence generation, allocation concealment, double-blinding, incomplete outcome data, selective reporting, and other bias. Studies that had a high, low, or unclear risk of bias for any of these six components were classified as high or low quality. The quality assessment of the retrospective comparative cohort study was performed using the Newcastle-Ottawa scale.

### Statistical Analysis

Data analyses were performed in Review Manager 5.4 software (version 5.4, The Cochrane Collaboration, The Nordic Cochrane Centre, Copenhagen, Denmark). Continuous variables were analyzed using mean differences (MDs) and 95% CIs. The Cochrane *Q* test and the *I*^2^ statistic were used to analyze the heterogeneity of the included studies, and *p* < 0.10 or *I*^2^ > 50% represented significant heterogeneity. We used a random effects model for the data analysis (*Q*-statistic: *P* < 0.10; *I*^2^ > 50%), or fixed effects model meta-analyses were performed (*Q*-statistic: *P* > 0.10; *I*^2^ < 50%). The overall result is statistically significant with a two-sided *p* < 0.05.

## Results

### Main Results

**Figure 1:**
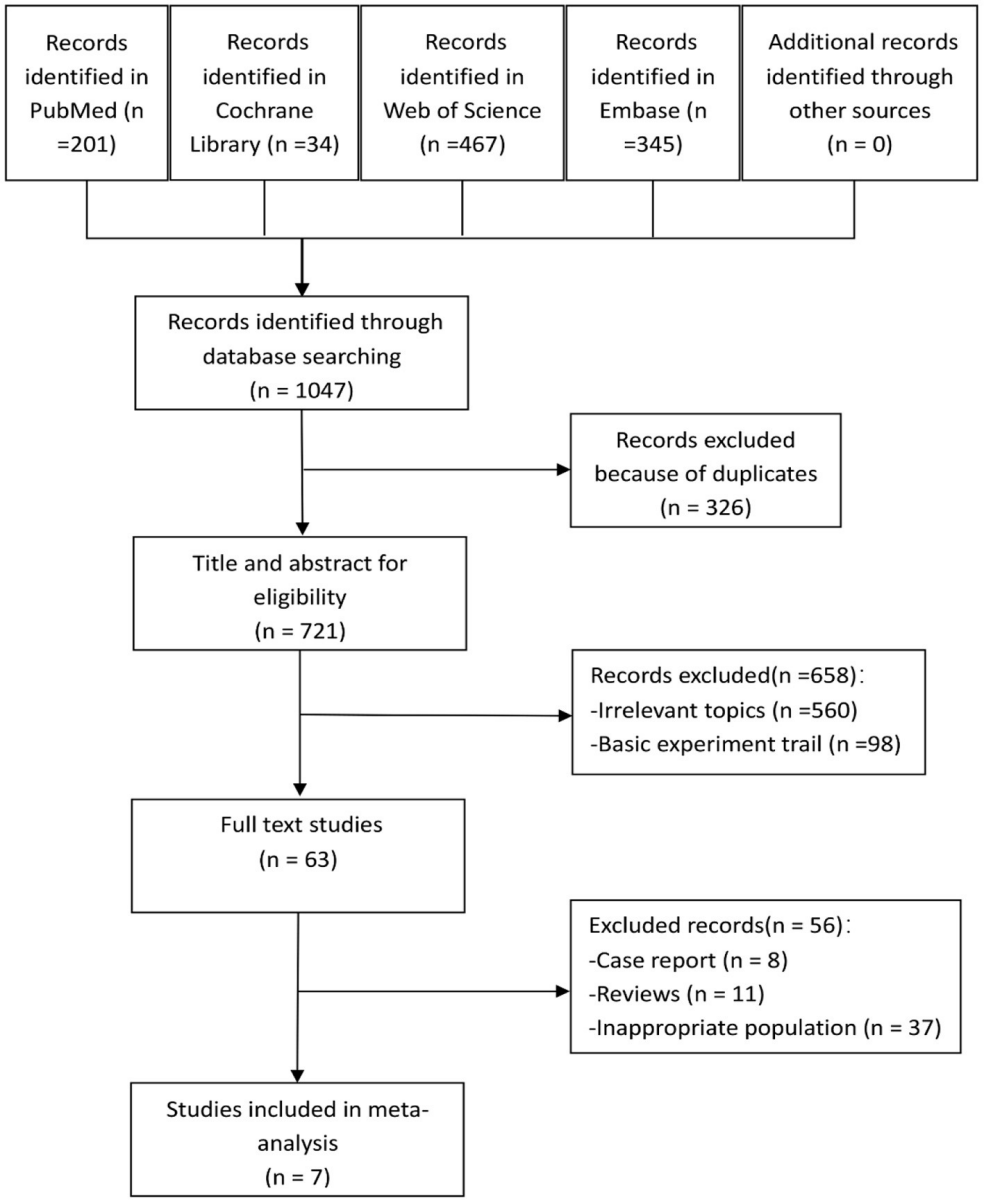
Flowchart of the studies included.

A total of 1,047 articles were found in our literature search, including 201 from PubMed, 34 from Cochrane Library, 467 from Web of Science, and 345 from Embase. Using endnote software, 326 repetitive studies were removed. After the selection of titles and abstracts, 658 studies were excluded, and the remaining 63 articles were screened for full text. After screening, seven studies met our inclusion criteria and 56 were excluded, six of which were RCTs (11–16), and one was a retrospective comparative control study (17). The study selection process is shown in Figure 1.

### Description of Included Studies

#### Characteristics

All the seven studies include 446 cases with a mean age of 6–13.6 years. The trials were designed to assess the changes index in complete remissions, serum albumin, serum creatinine, proteinuria, and eGFR. The treatment follow-up period was 12–48 months, and most trials included adverse events associated with childhood SDNS. The basic characteristics of the included studies are listed in Table 1. The interventions of treatment groups and control groups are clearly described in Table 2.

**Table 1:** Characteristics of studies included.

**References**	**Year,** **country**	**Study design**	**Group**	**Number**	**Age** **(years)**	**Sex** **(male/female)**	**The follow-up (months)**	**Event**
Ahn et al. (11)	2018, Korea	Multicenter RCT	RTX Control	35 16	13.5 (5.0) 12.5 (4.2)	26/9 13/3	12	Infusion reactions, such as chest discomfort, fever, vomiting, or skin rash
Basu et al. (12)	2018, India	Single-center, parallel RCT	RTX Control	60 60	7.1 (2.8) 7.2 (2.8)	32/28 32/28	12	Mild and transient transfusion reactions
Iijima et al. (13)	2014, Japan	Multicenter, double-blind RCT	RTX Control	24 24	11.5 (5.0) 13.6 (6.9)	18/6 16/8	12	Hypoproteinemia, lymphocytopenia, and neutropenia
Ravani et al. (14)	2011, Italy	Single-center, parallel RCT	RTX Control	27 27	10.2 (4.0) 11.3 (4.3)	24/3 19/8	12	Bronchospasm, hypotension, skin rash, and acute arthritis
Ravani et al. (15)	2020, Italy	RCT	RTX Control	15 15	7.4 (2.6) 6.0 (3.6)	9/6 12/3	48	Minimal skin rash and acute arthritis
Sinha et al. (17)	2012, India	Retrospective control comparative	RTX Control	10 13	12.2 (2.3) 12.3 (3.0)	8/2 10/3	12	Infusion reactions in the form of chills, myalgia, and transient skin rash
Solomon et al. (16)	2019, UK	Single-center, 2 parallel-arm RCT	RTX Control	60 60	–	–	12	–

**Table 2:** Interventions of studies included.

**References**	**Year, country**	**Interventions**
Ahn et al. (11)	2018, Korea	Treatment group: ① A single dose of intravenous RTX (375 mg/m^2^; maximum of 500 mg); ② steroids; and ③ calcineurin inhibitors. Control group: ① steroids and ② calcineurin inhibitors. (As long as remission was maintained, oral corticosteroids were reduced to 40 mg/m^2^ administered every other day for 4 weeks and then tapered by 25% every 4 weeks for 3 months, followed by calcineurin inhibitor tapering by 25% every 4 weeks).
Basu et al. (12)	2018, India	Treatment group: ① RTX two to four infusions at weekly intervals (375 mg/m^2^, maximum dose, 500 mg) and ② prednisolone. Control group: ① tacrolimus (0.2 mg/kg/day, targeting trough levels of 5 to 7 ng/ml) and ② prednisolone.
Iijima et al. (13)	2014, Japan	Treatment group: ① RTX an intravenous dose of 375 mg/m^2^ (maximum 500 mg) once weekly for 4 weeks; ② methyl prednisolone; ③ acetaminophen; and ④ d-chlorpheniramine maleate.Control group: ① prednisolone 60 mg/m^2^ orally 3 times a day (maximum of 80 mg/day) for 4 weeks, and then tapered over 6 weeks.
Ravani et al. (14)	2011, Italy	Treatment group: ① RTX (1 or 2 infusion of 375 mg/m^2^); ② intravenous chlorfenamine maleate; ③ methyl prednisolone; ④ oral paracetamol; ⑤ prednisone was tapered off by 0.3 mg/kg per week if proteinuria was <1 g/day; and calcineurin inhibitors. Control group: ① prednisone and calcineurin Inhibitors (doses of these agents could be tapered off as in the intervention strategy if proteinuria was <1 g/day).
Ravani et al. (15)	2020, Italy	Treatment group: ① RTX (1 infusion of 375 mg/m^2^); ② intravenous chlorfenamine maleate; ③ methyl prednisolone; ④ oral paracetamol; ⑤ prednisone (tapered off by 0.3 mg/kg/week starting at 30 days and withdrawn if proteinuria levels were still <1 g/m^2^/day). Control group: ① prednisone (tapered off by 0.3 mg/kg/week starting at 30 days and withdrawn if proteinuria levels were still <1 g/m^2^/day).
Sinha et al. (17)	2012, India	Treatment group: ① RTX (2 or 3 infusions of 375 mg/m^2^); ② tacrolimus (oral at a dose of 0.1–0.2 mg/kg/day in 2 divided doses for 12 months; ③ prednisolone (1.5 mg/kg on alternate days for 4 weeks, then reduced by 0.25 mg/kg every 2–4 weeks). Control group: ① tacrolimus (oral at a dose of 0.1–0.2 mg/kg/day in 2 divided doses for 12 months; ② prednisolone (1.5 mg/kg on alternate days for 4 weeks, then reduced by 0.25 mg/kg every 2–4 weeks).
Solomon et al. (16)	2019, UK	Treatment group: ① RTX 2 to 4 infusions (1/week, dose 375 mg/m^2^, maximum 500 mg), until B-cell depletion and ② prednisolone. Control group: ① tacrolimus: 0.2 mg/kg/day (target trough levels of 5–7 ng/ml) and ② prednisolone. (Both arms had tapering doses of alternate-day prednisolone over 12 months).

#### The Quality of the Studies

**Figure 2:**
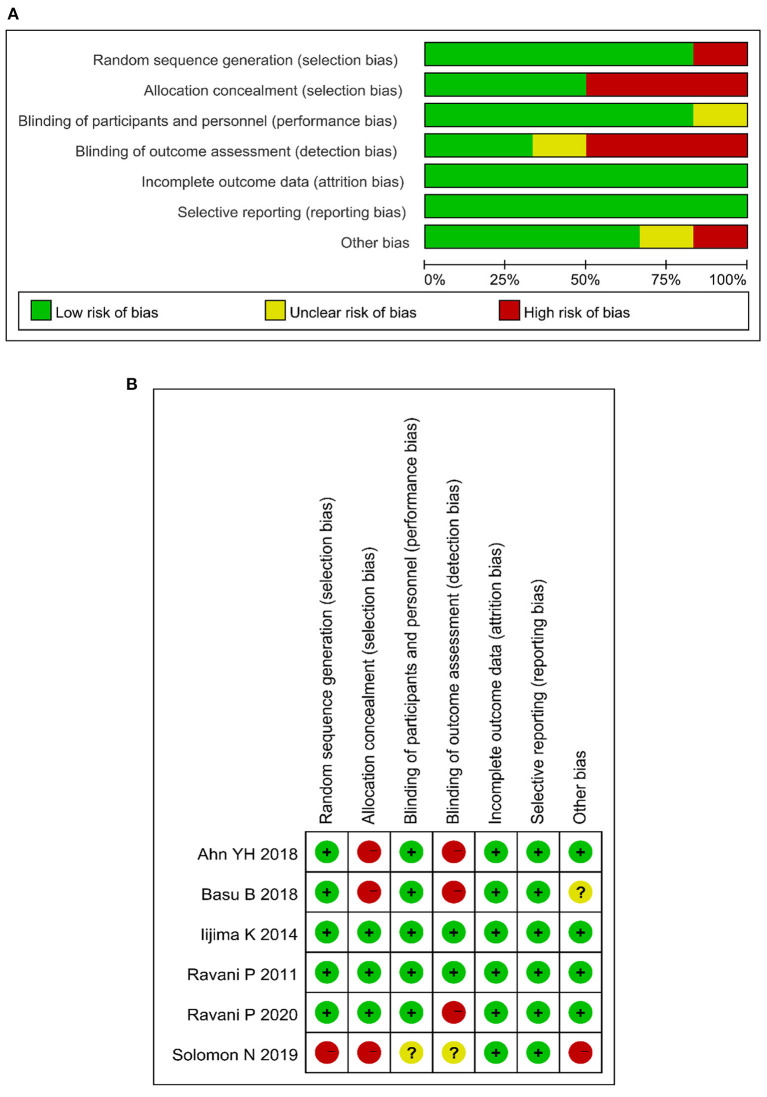
**(A)** Risk of bias graph. **(B)** Risk of bias summary.

The risk of bias in each RCT study was assessed by investigating random sequence generation, allocation concealment, blinding, integrity of outcome data, and the possibility of selective reporting (Figures 2A,B). The Newcastle-Ottawa scale scores awarded seven stars for a retrospective comparative control study reported by Sinha A (17).

### Efficacy of RTX in Childhood Steroid-Dependent Nephrotic Syndrome

#### Complete Remission Rate

**Figure 3:**
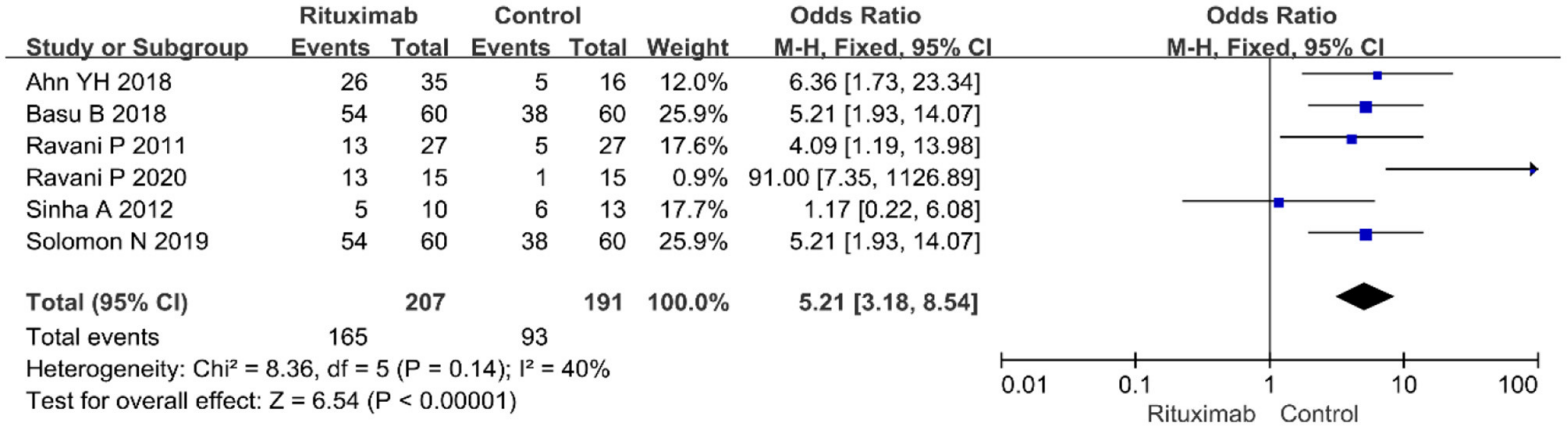
Forest plot showing a meta-analysis of complete remission rate between rituximab treatment group and control group.

Six studies reported the complete remission rates. The pooled data from these six studies indicate that RTX treatment group have a higher complete remission rate than control group (OR = 5.21; 95% CI, 3.18–8.54; *p* < 0.00001) appears in Figure 3.

#### Serum Albumin

**Figure 4:**
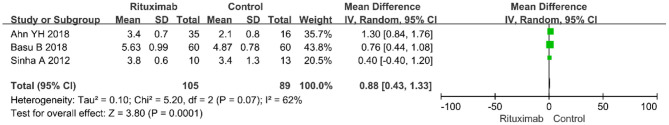
Forest plot showing a meta-analysis of serum albumin between rituximab treatment group and control group.

Three studies evaluated the results of serum albumin index (MD = 0.88; 95% CI, 0.43–1.33; *p* = 0.0001; *I*^2^ of 62% indicating heterogeneity) (Figure 4). Compared with the control group, RTX group had higher value of serum albumin.

#### Serum Creatinine

**Figure 5:**
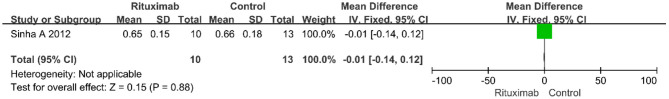
Forest plot showing a meta-analysis of serum creatinine between rituximab treatment group and control group.

Sinha A 2012 reported the result of serum creatinine and there was no significant difference between the two groups (MD = −0.01; 95% CI, −0.14 to 0.12; *p* = 0.88), as shown in Figure 5.

#### Proteinuria

**Figure 6:**
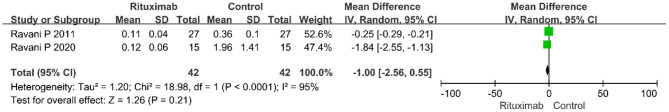
Forest plot showing a meta-analysis of proteinuria between rituximab treatment group and control group.

Two studies evaluated the results of proteinuria level, and there was no significant difference between the two groups (MD = −1.00; 95% CI, −2.56 to 0.55; *p* = 0.21; *I*^2^ of 95% indicating heterogeneity), as shown in Figure 6.

#### eGFR

**Figure 7:**
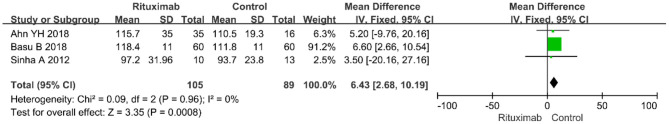
Forest plot showing a meta-analysis of eGFR between rituximab treatment group and control group.

Three studies reported the results of eGFR (MD = 6.43; 95% CI, 2.68–10.19; *p* = 0.0008; *I*^2^ of 0% indicating no heterogeneity) (Figure 7). Compared with the control group, RTX group had higher value of eGFR.

### Safety

#### Infections

**Figure 8:**
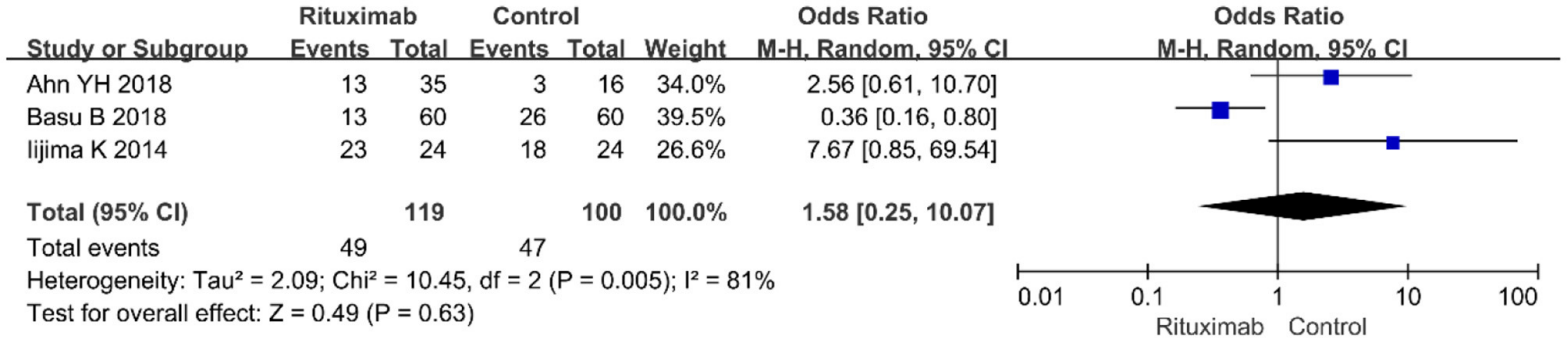
Forest plot showing a meta-analysis of infections between rituximab treatment group and control group.

Three studies reported the infections (OR = 1.58; 95% CI, 0.25–10.07; *p* = 0.63; *I*^2^ of 81% indicating heterogeneity) (Figure 8), and no significant differences were observed in the occurrence rate of infections between the two groups.

#### Infusion Reactions

**Figure 9:**
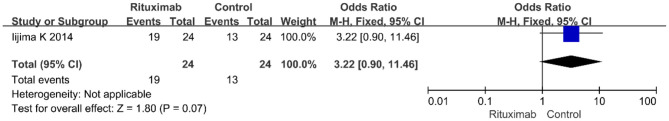
Forest plot showing a meta-analysis of infusion reactions between rituximab treatment group and control group.

Iijima et al. (13) reported the results of infusion reaction events (OR = 3.22; 95% CI, 0.90–11.46; *p* = 0.07) (Figure 9), and no significant differences were observed in the occurrence rate of infusion reactions between the two groups.

#### Cardiovascular Disease Events

**Figure 10:**
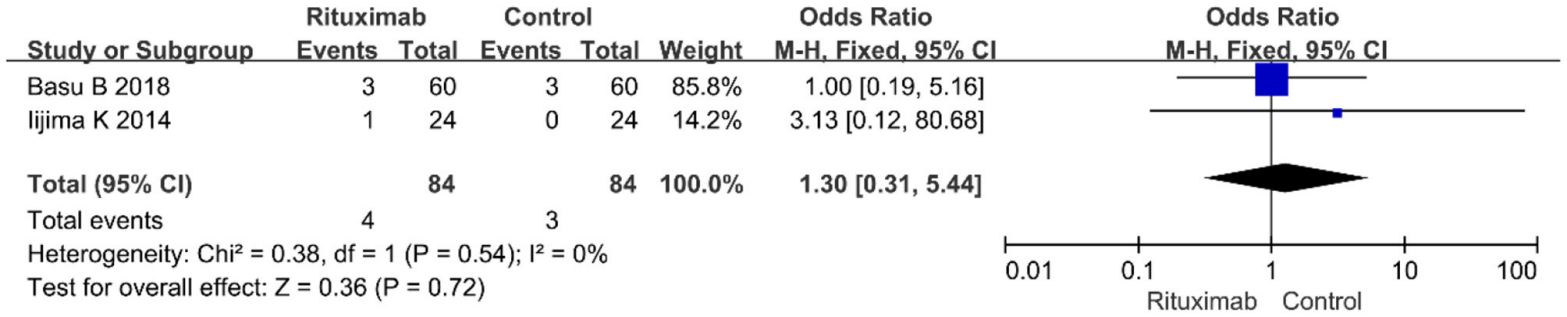
Forest plot showing a meta-analysis of cardiovascular disease events between rituximab treatment group and control group.

Two studies reported the results of cardiovascular disease events (OR = 1.30; 95% CI, 0.31–5.44; *p* = 0.72; *I*^2^ of 0% indicating no heterogeneity) (Figure 10), and no significant differences were observed in the occurrence rate of cardiovascular disease events between the two groups.

## Discussion

Our meta-analysis included six RCTs and one retrospective comparative control study, involving a total of 446 patients, including 231 in the rituximab group and 215 in the control group. Data analysis results showed that compared with the control group, the RTX treatment group can significantly improve the complete remission rate of children with SDNS. In addition, the RTX treatment group is better than the control group in improving the level of serum albumin and estimated glomerular filtration rate, and the difference is statistically significant. The results also suggest that the RTX treatment group had a better outcome in reducing the occurrence of proteinuria than the control group, but the difference is not statistically significant. There were no significant differences in serum creatinine levels and related adverse events between the two groups.

The conventional view is that disproportion, activity alterations, and regulatory cytokines of T cell are involved in the pathogenesis of childhood nephrotic syndrome. In addition, B cells can enhance T-cell responses by producing antibodies, stimulatory cytokines, producing inflammatory responses, thereby accelerating and aggravating the occurrence and development of NS (18). RTX is a novel therapeutic approach to the clinical management of NS, which induces cell apoptosis by binding to the CD20 membrane antigen in both normal and malignant B cells (19, 20). However, there are also studies that propose NS is treatable by podocyte-specific expression of SMPDL3b in RTX therapy (21).

RTX showed an advantage in the complete remission rate, which is consistent with previous related research findings. A study analyzed 33 patients of childhood SDNS treatment with RTX, and the remission rates were 48.5% after 6 months, among which most were sustained remission (15 cases, 94%) (22). Prytula et al. reported that ~66% of patients achieved complete or partial remission after RTX therapy (23). Another study reported that 20 (80%) of 25 SDNS patients achieved complete remission after receiving RTX treatment, and one of the remaining cases has recurred after withdrawal of RTX (24). Indeed, these data showed that RTX treatment can be effective in achieving complete remission.

RTX could effectively improve eGFR level and serum albumin level in patients with childhood SDNS. Some studies (11, 12, 25) have also confirmed the long-term benefits or risks of the use of this new type of drug compared with the usual immunosuppressive agents in the past. Therefore, RTX can be used to improve the serum albumin and eGFR levels of patients, which facilitate the remission and recovery of the disease.

However, RTX neither significantly decreased the levels of serum creatinine nor significantly reduced the occurrence of proteinuria, and the results were inconsistent with data published now. The discrepancy may be due to the following reasons. First, the differences of pathological types existed in different patients, which varied in their response to RTX treatment. Second, the usage and dosage of RTX were not uniform in the included study, and this may also have an impact on the results. Third, only one study presented the data on serum creatinine among all the included studies, so the number of included cases was small and therefore not representative.

RTX were well-tolerated in most patients of NS, with infusion reactions as the most frequently reported adverse effect, and the incidence was 5–53% (26). Generally, slowing down the rate of infusion or applying antihistamines can alleviate it, and very few children will have severe allergic reactions (27). In this study, RTX treatment did not cause a significant reduction on the incidence of adverse events and did not show an advantage on safety (11–13). However, when some patients cannot tolerate immunosuppressive agents or develop drug resistance to these immunosuppressive agents, RTX treatment may lead to a positive curative effect (5).

However, there are still a few limitations in this study. First, studies included in our meta-analysis enrolled patients from different regions or countries, with different symptoms, and there are some basic characteristic differences among these patients, in addition, the follow-up duration of these studies was not unified; all of these factors may result in some of the heterogeneity in some of our results. Second, only six RCTs and one retrospective comparative control study were included in the meta-analysis; the number was small and with insufficient clinical evidence, which may result in some statistical bias or error and could reduce the evaluation power. Third, there were different rituximab therapy regimens used in the included studies, while both rituximab dose and maintenance immunosuppression have important effects on the treatment outcomes (28), so it may have had an impact on our analysis results. Fourth, studies included in our meta-analysis had different control groups, which might influence the results of our analysis. Fifth, the number of included cases was relatively small and thus may be underrepresented in the study sample. In addition, RTX treatment is a high-cost therapy, but the relationship between the costs and efficacy of this drug did not reflect in these included studies. Thus, further studies are needed to refine these issues.

## Conclusions

In conclusion, RTX can be considered a safe and efficient alternative therapy for childhood SDNS. Steroid achieves remission, while RTX plays a role in SDNS by maintaining remission, avoiding relapse, and avoiding further steroid therapy. RTX not only has more advantages in complete remission rate than other immune suppressants but also has ameliorative effects on eGFR and serum albumin. However, the safety and long-term efficacy of RTX have not been fully evaluated, therefore future studies with higher quality, larger sample sizes, and longer durations of follow-up are needed to address this question.

## Data Availability Statement

The original contributions presented in the study are included in the article/supplementary material, further inquiries can be directed to the corresponding author/s.

## Author Contributions

SL: conceptualization, writing (original draft), methodology, and software. CG: writing (review and editing). ZL: formal analysis. HL: investigation and data curation. ZF: investigation and data curation. YD: writing (review and editing), supervision, and funding acquisition. All authors contributed to the article and approved the submitted version.

## Conflict of Interest

The authors declare that the research was conducted in the absence of any commercial or financial relationships that could be construed as a potential conflict of interest.

## Publisher's Note

All claims expressed in this article are solely those of the authors and do not necessarily represent those of their affiliated organizations, or those of the publisher, the editors and the reviewers. Any product that may be evaluated in this article, or claim that may be made by its manufacturer, is not guaranteed or endorsed by the publisher.
